# Biography of Contemporary Physicians of Texas

**Published:** 1886-11

**Authors:** 


### BIOGRAPHY OF CONTEMPORARY PHYSICIANS OF TEXAS,

EDITED BY F. E. DANIEL, M. D.

The canvas for this work, which has been interrupted somewhat by the hard times and scarcity of money, is now renewed, and Messrs. S. H. Dixon & Co., the publishers who have the contract for collecting the data, and for publishing it in accordance with the Prospectus originally announced, inform us that it will be pushed to completion at as early day as practicable. It is, however, a work that will require time, and much labor and care; and as there is no necessity for hurry, there will be none. Subscribers must rest patient; and meanwhile the list is open; and only room for a few more portraits. Better terms have been made for engraving from photographs, and we are authorized to say they will be inserted for $20, instead of $35, as announced. Address S. H. Dixon & Co., Austin, Texas.

Mr. Dixon informs me that he has the subscription and portrait of most of the Presidents of the State Association, and many other older practitioners. The portraits of our recently deceased brothers, Drs. Ashbell Smith, W. J. Burt and W. H. Parks, will be inserted, with the obituary notices, as well as their biography. Lose no time in securing your space, or you will regret it.

*Wanted*—Experienced canvassers; liberal compensation, and a paying job.

